# One episode of low intensity aerobic exercise prior to systemic AAV9 administration augments transgene delivery to the heart and skeletal muscle

**DOI:** 10.1186/s12967-023-04626-1

**Published:** 2023-10-24

**Authors:** Christina A. Pacak, Silveli Suzuki-Hatano, Fatemeh Khadir, Audrey L. Daugherty, Mughil Sriramvenugopal, Bennett J. Gosiker, Peter B. Kang, William Todd Cade

**Affiliations:** 1 Paul and Sheila Wellstone Muscular Dystrophy Center and Department of Neurology, University of Minnesota Medical School, 420 Delaware St SE, Minneapolis, MN 55455 USA; 2 College of Medicine, Department of Pediatrics, University of Florida, Gainesville, USA; 3 Physical Therapy Division, Department of Orthopaedic Surgery, Duke University School of Medicine, 311 Trent Drive, Durham, NC 27710 USA

**Keywords:** Gene therapy, Systemic gene delivery, Adeno-associated virus, AAV, AAV9, Aerobic exercise, Cardioskeletal myopathy, Barth syndrome, *TAFAZZIN*

## Abstract

**Introduction:**

The promising potential of adeno-associated virus (AAV) gene delivery strategies to treat genetic disorders continues to grow with an additional three AAV-based therapies recently approved by the Food and Drug Administration and dozens of others currently under evaluation in clinical trials. With these developments, it has become increasingly apparent that the high doses currently needed for efficacy carry risks of toxicity and entail enormous manufacturing costs, especially for clinical grade products. Strategies to increase the therapeutic efficacy of AAV-mediated gene delivery and reduce the minimal effective dose would have a substantial impact on this field. We hypothesized that an exercise-induced redistribution of tissue perfusion in the body to favor specific target organs via acute aerobic exercise prior to systemic intravenous (IV) AAV administration could increase efficacy.

**Background:**

Aerobic exercise triggers an array of downstream physiological effects including increased perfusion of heart and skeletal muscle, which we expected could enhance AAV transduction. Prior preclinical studies have shown promising results for a gene therapy approach to treat Barth syndrome (BTHS), a rare monogenic cardioskeletal myopathy, and clinical studies have shown the benefit of low intensity exercise in these patients, making this a suitable disease in which to test the ability of aerobic exercise to enhance AAV transduction.

**Methods:**

Wild-type (WT) and BTHS mice were either systemically administered AAV9 or completed one episode of low intensity treadmill exercise immediately prior to systemic administration of AAV9.

**Results:**

We demonstrate that a single episode of acute low intensity aerobic exercise immediately prior to IV AAV9 administration improves marker transgene delivery in WT mice as compared to mice injected without the exercise pre-treatment. In BTHS mice, prior exercise improved transgene delivery and additionally increased improvement in mitochondrial gene transcription levels and mitochondrial function in the heart and gastrocnemius muscles as compared to mice treated without exercise.

**Conclusions:**

Our findings suggest that one episode of acute low intensity aerobic exercise improves AAV9 transduction of heart and skeletal muscle. This low-risk, cost effective intervention could be implemented in clinical trials of individuals with inherited cardioskeletal disease as a potential means of improving patient safety for human gene therapy.

## Introduction

A unifying aim for investigations of recombinant adeno-associated virus (AAV) mediated therapeutic strategies is to identify synergistic approaches to reduce the minimal effective dose (MED) and optimize therapeutic effectiveness. Successful dose reduction improves patient safety, increases the number of patients treated with each product lot, and reduces prices for these treatments, which are among the costliest in the world. Previously validated approaches to improve the efficiency of AAV strategies include optimization of promoter and enhancer sequences, the use of double-stranded (dsAAV) vectors when feasible, selection of an AAV capsid with high tropism for the target tissues, and precision-guided delivery techniques [1–5]. While each of these represent key improvements to AAV-mediated gene therapies, there is an urgent need for further advances to improve overall efficacy.

Localized AAV injections are sufficient for certain targets (e.g., retina). However, many inherited disorders affect multiple tissues and organs (e.g., heart and skeletal muscle), requiring systemic delivery approaches to distribute the vector throughout the target(s) [6]. Systemic AAV delivery is complicated by factors such as vector genome (vg) uptake by non-target tissues and vector waste due to ubiquitination and autophagic degradation pathway activation following endocytosis, leading to the current need for high intravenous (IV) doses of AAV [7]. Counter-productively, higher systemic doses carry an increased risk of eliciting immune responses that can cause direct and immediate harm to patients and risk negating the therapeutic benefits [8].

We hypothesized that an exercise-induced redistribution of tissue perfusion in the body to favor specific target organs via acute aerobic exercise prior to systemic intravenous (IV) AAV administration could increase AAV vector genome (vg) uptake and improve AAV efficacy in the heart, muscle. Exercise mediates a myriad of physiologic and molecular responses in numerous tissues in the body including skeletal muscle, heart, liver, vasculature, gut, brain, and immune system [9–11]. Sustained muscle contractions during exercise increase blood flow and tissue perfusion linearly with increasing exercise intensity levels through increased cardiac output, shunting of cardiac output from non-exercising organs, and local vasodilation of arterioles and capillaries [11–14]. Exercise also causes changes in circulating cytokine profiles that influence a wide variety of signaling pathways [15–19]. We were interested in determining whether these exercise-induced changes could augment skeletal muscle and cardiac transduction of systemically-administered AAV in a safe, non-invasive, inexpensive manner that would be simple to implement in a clinical setting.

We therefore designed a set of experiments to examine whether low intensity exercise sufficient to increase heart and skeletal muscle perfusion would improve intravenously administered AAV9 transduction in wild type mice and a mouse model of Barth Syndrome (BTHS). BTHS is a rare, X-linked, mitochondrial disorder that leads to severe cardioskeletal myopathy and is caused by mutations in the gene *TAFAZZIN* that encodes tafazzin—an acyltransferase in mitochondria that remodels cardiolipin [20–22]. Although we have previously demonstrated the benefits of gene therapy for BTHS in mice and cells, here we demonstrate a therapeutic effect using a new human codon optimized TAFAZZIN vector being proposed for clinical evaluation [6, 23, 24]. We also demonstrate here that one round of acute low intensity aerobic exercise immediately prior to systemic administration of AAV9-Des-co*TAFAZZIN* improves vector genome (vg) uptake, increases transcription levels, and improves mitochondrial function in the heart and skeletal muscle.

## Results

**Fig. 1:**
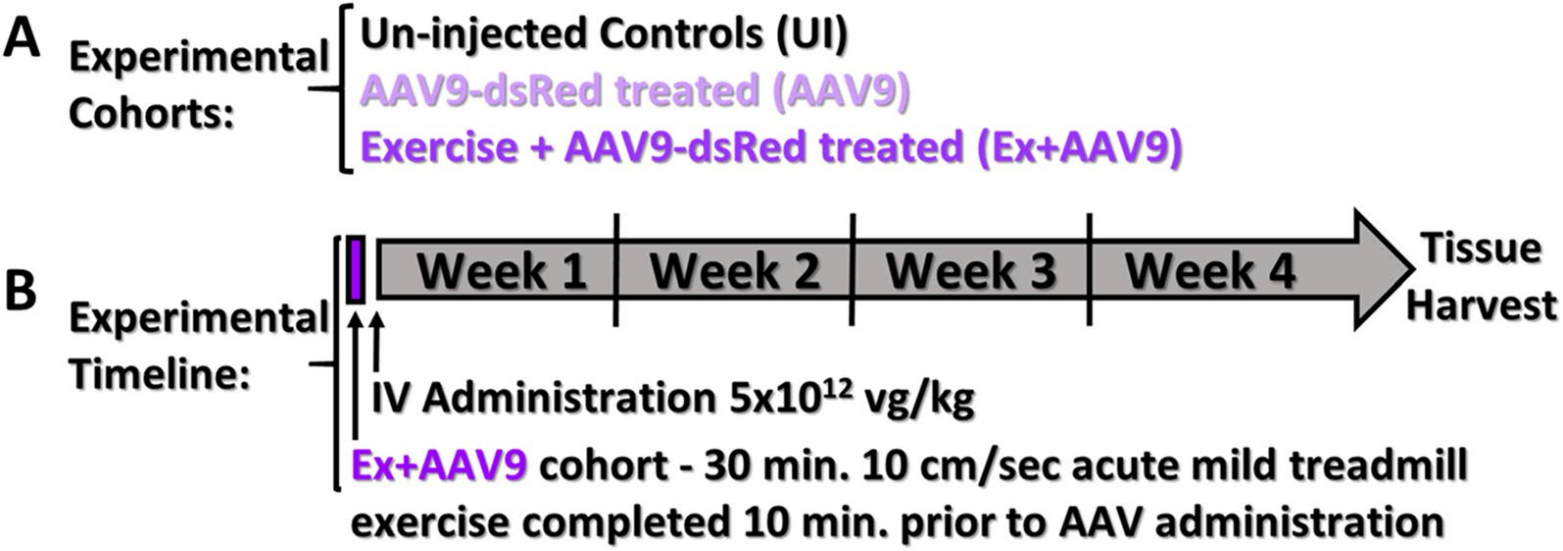
**A** Experimental cohorts, and **B** experimental timeline with exercise description and AAV9 administration indicated. Mice were systemically administered AAV9 via the jugular vein as soon as the animals were anesthetized and prepared for IV injections. At four weeks post-administration, mice were euthanized and tissues harvested

To study the effect of aerobic exercise immediately prior to intravenous (IV) AAV9 administration on transduction across multiple tissue types, a marker gene based comparative study was performed. We compared A) uninjected (UI) healthy C57BL/6 mice to B) mice of the same strain injected with 5 × 10^12^ vg/kg of a double-stranded AAV9 (dsAAV9) construct containing a CMV promoter driving expression of a mitochondrial dsRed marker gene (CMV-dsRed) via jugular vein IV administration without an exercise regimen (AAV9), and C) mice of the same strain injected with the same AAV9 as in (B), but with the addition of low intensity acute exercise (30 min of treadmill running at 10 cm/sec) immediately prior to administration (Ex + AAV9) (Fig. 1A, B) [2, 25, 26].

**Fig. 2:**
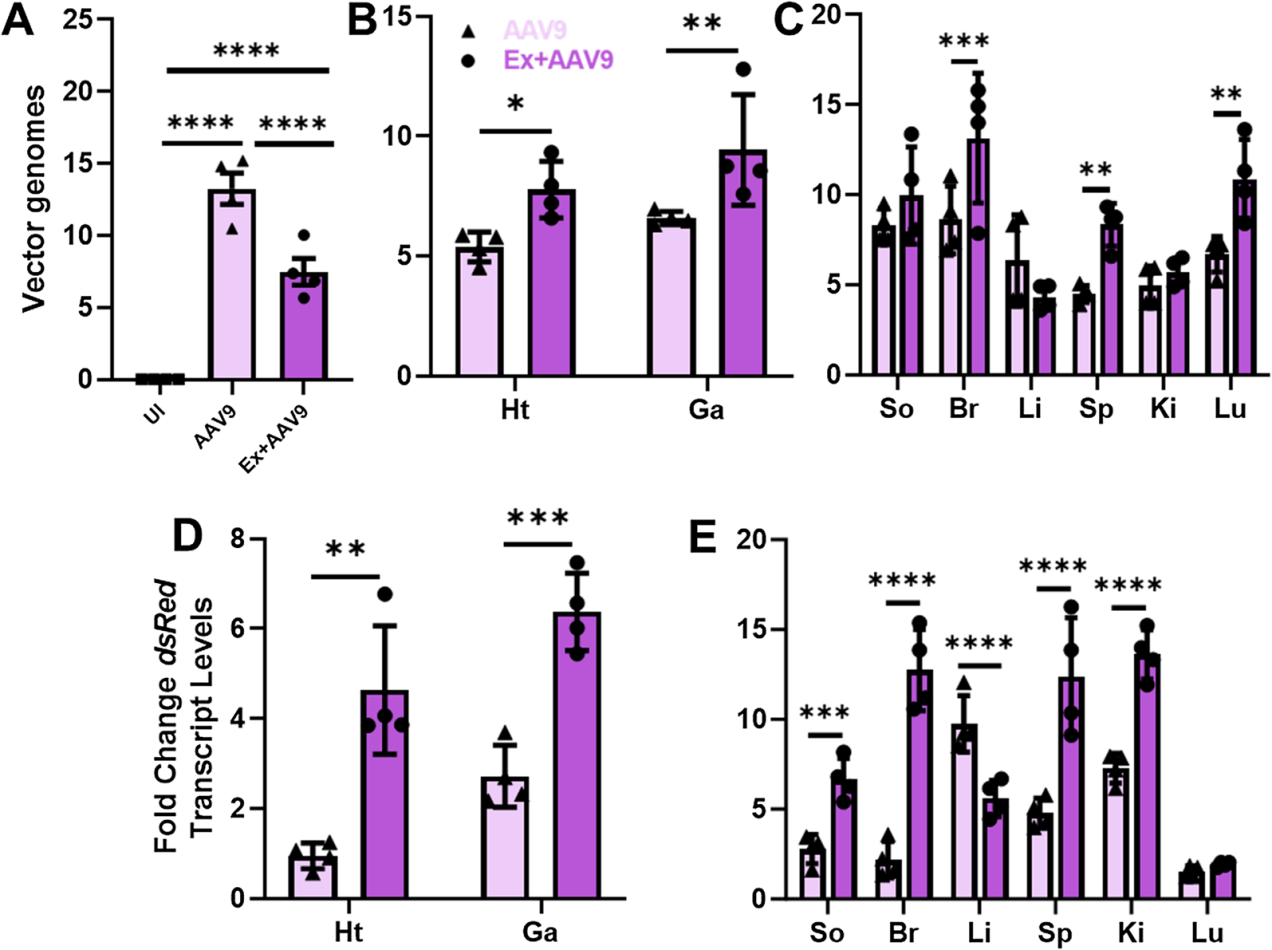
Vector genomes per nanogram (ng) total genomic DNA in un-injected (UI), AAV9 injected without exercise (AAV9), or exercised and then injected with AAV9 (Ex + AAV9) cohorts of mice in **A** whole blood, **B** heart (Ht) and gastrocnemius (Ga), and **C** other tissues soleus (So), brain (Br), liver (Li), spleen (Sp), kidney (Ki), and lung (Lu). Fold increase in CMV-dsRed transgene transcript levels in **D** Ht and Ga, and **E** other tissues as compared to uninjected controls. (t-tests were performed for comparisons between AAV9 and Ex + AAV9 cohorts and data indicate mean ± SEM) (n = 4) *p ≤ 0.05, **p ≤ 0.01, ***p ≤ 0.005, ****p ≤ 0.001

At four weeks post-injection, mice were euthanized, tissues and blood collected, and vector genome (vg) distribution measured. A significant decrease in vgs identified were observed in whole blood isolated from the exercised cohort (Ex + AAV9) as compared to the non-exercised cohort (AAV9) suggesting reduced circulating vgs (Fig. 2A). In contrast, evaluations of vgs in heart (Ht) and gastrocnemius (Ga) showed a significant increase in uptake in both tissues for the Ex + AAV9 mice as compared to the AAV9 mice (Fig. 2B). Of the other tissues assessed, brain (Br), spleen (Sp), and lung (Lu) also showed a significant increase in vg uptake in Ex + AAV9 mice as compared to AAV9 mice while soleus, liver, and kidney did not (Fig. 2C). Evaluations of fold change in transcript levels also revealed significant increases in both heart and gastrocnemius from the Ex + AAV9 mice as compared to the AAV9 mice (Fig. 2D) and results from other tissues generally mimicked the trends observed from vg results (Fig. 2E).

**Fig. 3:**
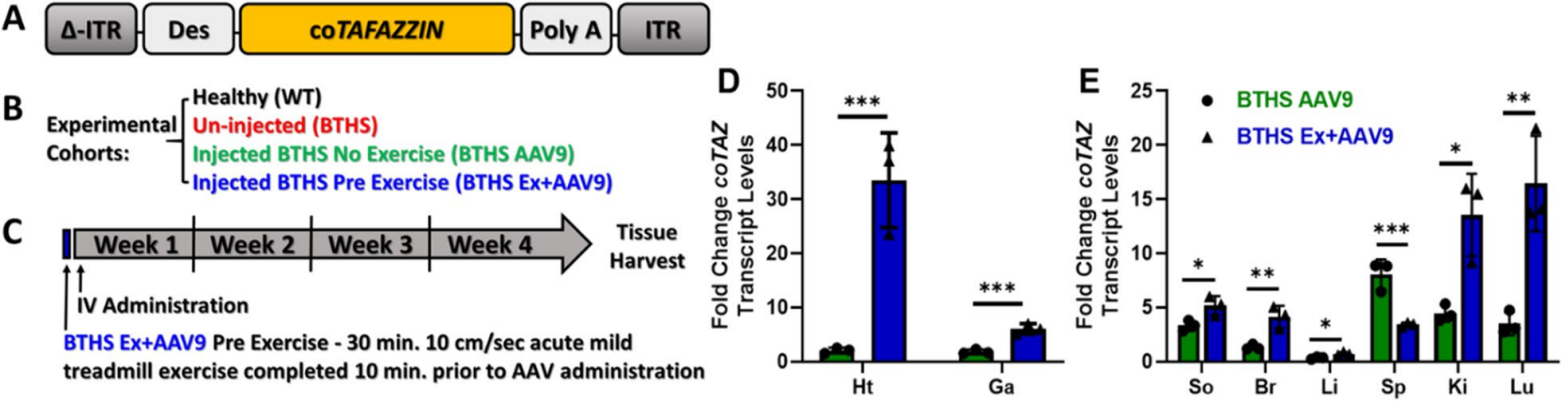
BTHS mouse gene therapy experimental setup showing **A** the therapeutic vector design, **B** the experimental cohorts, and **C** the experimental timeline. **D** Graph depicting the fold increase in co*TAFAZZIN* transcript levels over untreated BTHS controls in heart and gastrocnemius. **E** Fold increase in co*TAFAZZIN* transcripts over untreated controls in other tissues. Heart (Ht), gastrocnemius (Ga), soleus (So), brain (Br), liver (Li), spleen (Sp), kidney (Ki), lung (Lu). (t-tests were performed for comparisons between BTHS AAV9 and BTHS Ex + AAV9 cohorts and data indicate mean ± SEM) *p ≤ 0.05, **p ≤ 0.01, ***p ≤ 0.005

We then tested whether or not the acute, low-intensity exercise approach could be incorporated into our gene therapy strategy for BTHS. We compared healthy adult WT mice (WT), un-injected BTHS mice (BTHS), BTHS mice treated with a systemic IV administration of 5 × 10^12^ vg/kg of a dsAAV9-Des-co*TAFAZZIN* (human codon optimized *TAFAZZIN*) vector with no exercise (BTHS AAV9), and BTHS mice provided the same AAV9 treatment immediately following the same single episode of low intensity, acute aerobic exercise described above (BTHS Ex + AAV9) (Fig. 3A–C). At four weeks post-injection, tissues were collected and compared across cohorts. Acute exercise significantly increased transcription levels of the transgene *TAFAZZIN* in the heart and gastrocnemius (Fig. 3D) as well as soleus, brain, liver, kidney, and lung (Fig. 3E). While the spleen showed higher expression levels than other tissues in the BTHS AAV9 cohort, a significant decrease was found in the BTHS Ex + AAV9 (Fig. 3E).

**Fig. 4:**
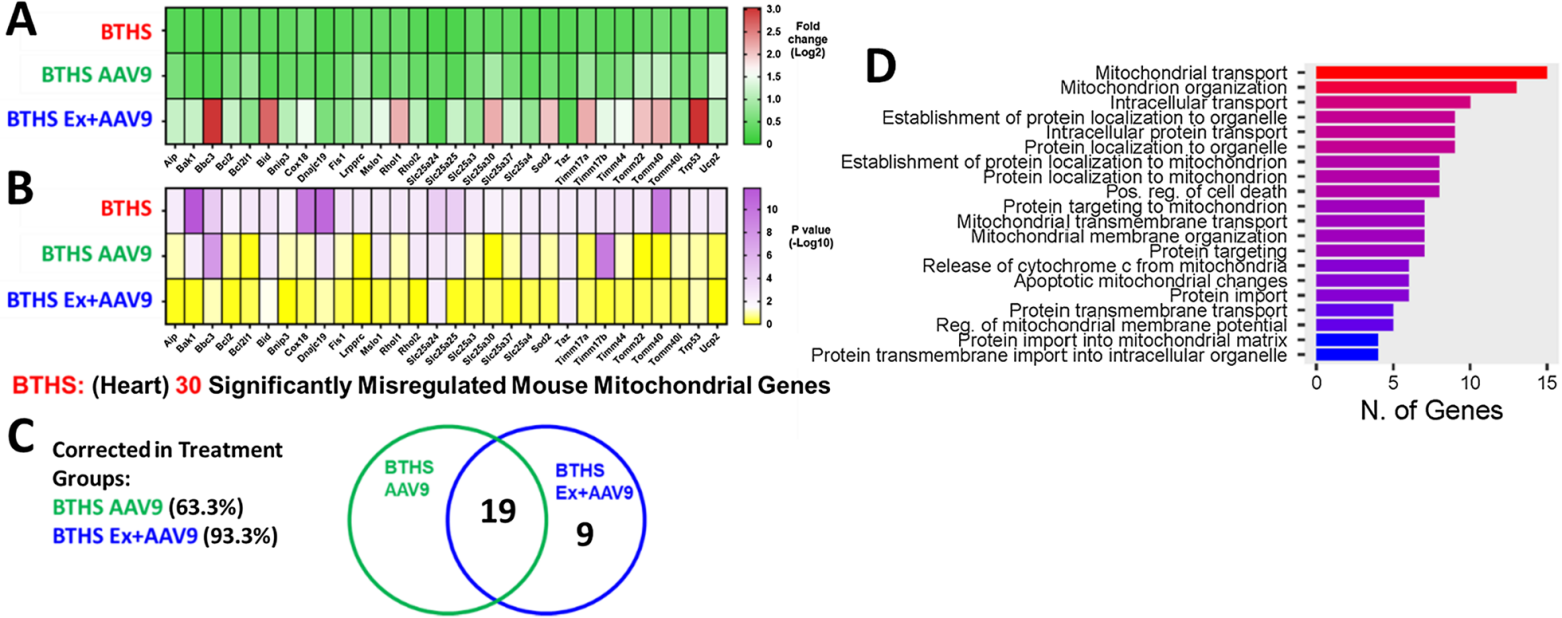
RT^2^ Profiler PCR Array Mouse Mitochondria Data (Heart). Heat-map displays of all 30 transcripts found to be significantly altered between BTHS and WT samples amongst the 84 assessed in the mitochondrial transcription array. **A** Heat map (green/red) display of average fold change for each transcript as compared to WT controls with each cohort in a separate row. **B** Heat map (yellow/purple) -Log10 p-values depicting the extent of significant difference for each cohort in a separate row as compared to WT controls. A total of 30 genes were found to be significantly downregulated in the BTHS heart as compared to age-matched healthy controls. **C** Venn diagram depiction of the number of genes that were corrected in the respective treatment groups. **D** A bar plot showing number of genes involved in the listed biological processes generated with ShinyGO 0.77 using the list of 19 genes that were corrected in both treatment groups. Profile arrays were run in quadruplicate for each of 3 samples from each cohort. (p ≤ 0.05 was considered to be a significant difference as compared to WT)

Next, RT^2^ Profiler PCR arrays were used to assess expression levels of 84 mitochondrial genes. These showed that a total of 30 genes were significantly differentially expressed in untreated BTHS hearts as compared to healthy, age-matched WT controls. A significant difference was based upon a p-value ≤ 0.05 (Fig. 4A, B). 19 gene transcript levels were normalized in the hearts (no significant difference as compared to WT controls) of both the BTHS AAV9 and BTHS Ex + AAV9 cohorts (Fig. 4C). An additional 9 gene transcript levels were normalized in the hearts of only the BTHS Ex + AAV9 mice. The 19 genes that were corrected in both treatment groups were assessed using gene ontology (ShinyGO) analysis. Highly represented biological processes amongst this group of genes include mitochondrial transport and organization (Fig. 4D).

**Fig. 5:**
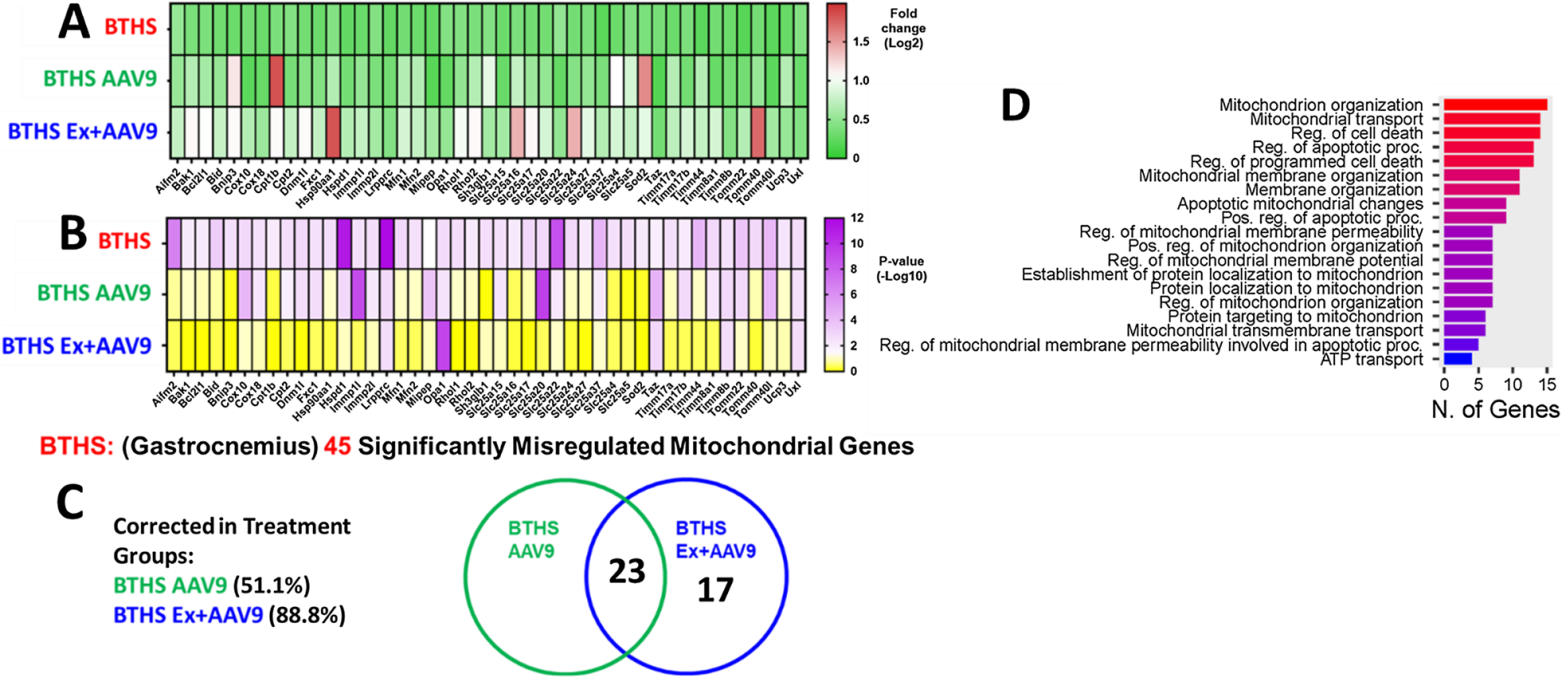
RT^2^ Profile PCR Array Mouse Mitochondria Data (Gastrocnemius). Heat-map displays of all 45 transcripts found to be significantly altered between BTHS and WT samples amongst the 84 assessed in the mitochondrial transcription array. **A** Heat-map (green/red) display of average fold change for each transcript with each cohort in a separate row as compared to WT controls. **B** Heat map (yellow/purple) -Log10 p-values depicting the extent of significant difference for each cohort in a separate row as compared to WT controls. A total of 45 genes were found to be significantly downregulated in the BTHS gastrocnemius as compared to age-matched healthy controls. **C** Venn diagram depiction of the number of genes that were corrected in the respective treatment groups. **D** A barplot showing number of genes involved in the listed biological processes generated with ShinyGO 0.77 using the list of 23 genes that were corrected in both treatment groups. Profile arrays were run in quadruplicate for each of 3 samples from each cohort. (p ≤ 0.05 was considered to be a significant difference as compared to WT)

The same RT^2^ Profiler PCR arrays were used to compare gastrocnemius samples from these cohorts and showed that a total of 45 genes from the array were significantly differentially expressed in untreated BTHS hearts as compared to healthy, age-matched WT controls (Fig. 5A, B). 23 gene transcript levels were normalized in the gastrocnemius (no significant difference as compared to WT controls) of both the BTHS AAV9 and BTHS Ex + AAV9 cohorts (Fig. 5C). An additional 17 gene transcripts were normalized in the gastrocnemius of only the BTHS Ex + AAV9 mice. The 23 genes that were corrected in both treatment groups were assessed using ShinyGO analysis. These results showed that highly represented biological processes amongst this group of genes include mitochondrial organization and transport and regulation of apoptosis and cell death (Fig. 5D).

**Fig. 6:**
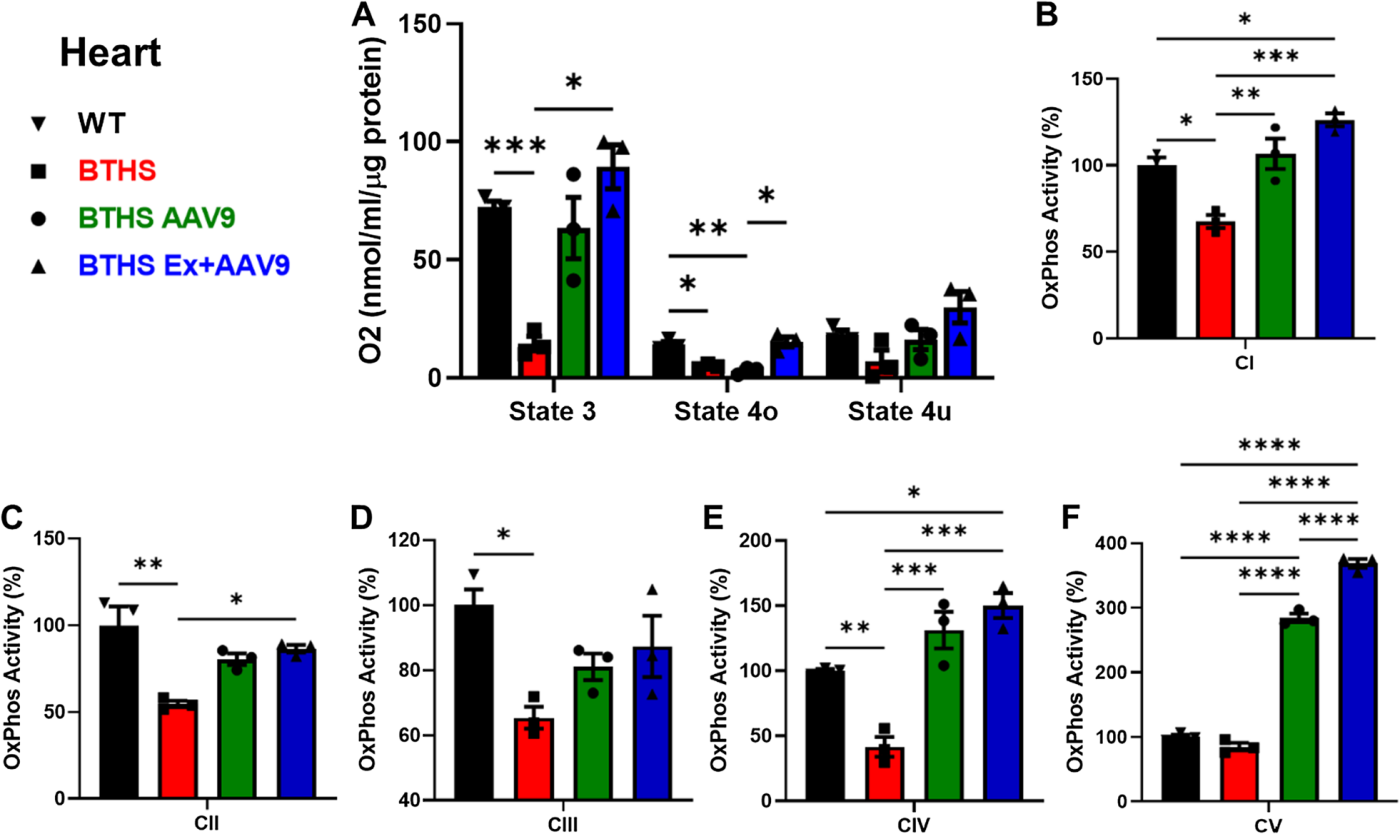
Functional analyses on mitochondria isolated from BTHS mouse hearts. **A** Oxygen consumption data. Enzyme activity data for ETC complexes I-V graphed as compared to WT (100%) for **B** complex 1, **C** complex 2, **D** complex 3, **E** complex 4, **F** complex 5. Comparisons were made using ANOVA *p ≤ 0.05,**p ≤ 0.01,***p ≤ 0.001,****p ≤ 0.0001

To evaluate whether increased *TAFAZZIN* expression levels in BTHS Ex + AAV9 mice hearts resulted in greater improvements in mitochondrial function, we first measured O_2_ consumption on isolated heart mitochondria from all cohorts. These experiments showed a significant decrease in States 3 and 4o O_2_ consumption in untreated BTHS mice compared to WT controls. BTHS Ex + AAV9 mice demonstrated a significant improvement in State 3 O_2_ consumption compared to BTHS, and a significant improvement in State 4o O_2_ consumption compared to BTHS AAV9 mice with no exercise (Fig. 6A). There were no significant differences between WT and BTHS Ex + AAV9 mice in any states measured.

Next, we performed assays to measure the enzyme activity levels of each of the five electron transport chain (ETC) complexes (CI, CII, CIII, CIV, and CV) in mitochondria isolated from the hearts of mice from all cohorts. A significant decrease in enzyme activity levels was found in CI-IV activities in mitochondria isolated from untreated BTHS hearts compared to healthy controls (Fig. 6B–F). BTHS AAV9 mice showed significant increases in CI, CIV, and CV activity levels as compared to BTHS and BTHS Ex + AAV9 mice showed significant increases in CI, CII, CIV, and CV activity levels compared to untreated BTHS controls.

**Fig. 7:**
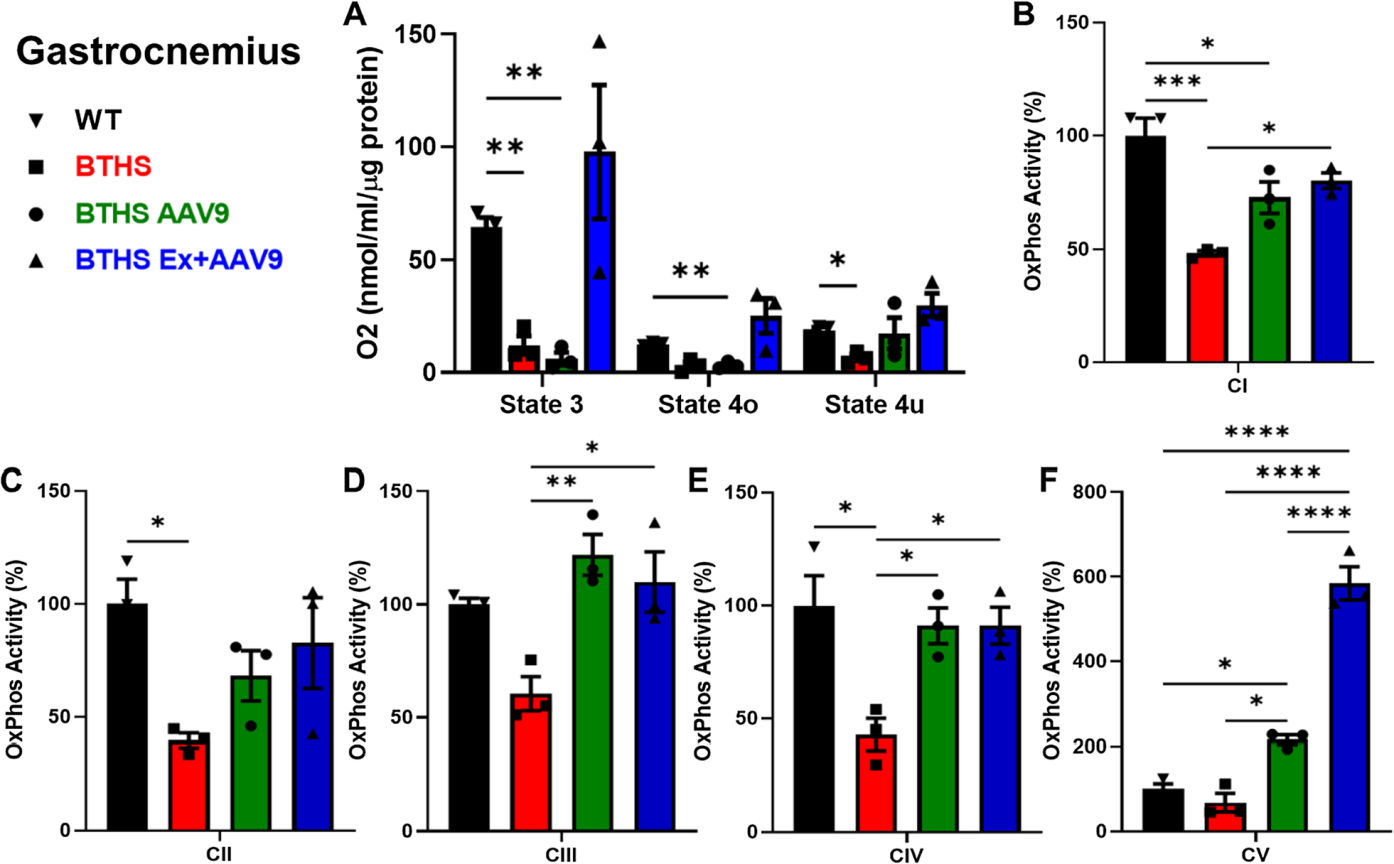
Functional analyses on mitochondria isolated from BTHS mouse gastrocnemius muscles. **A** Oxygen consumption data. Enzyme activity data for ETC complexes I-V graphed as compared to WT (100%) for **B** complex 1, **C** complex 2, **D** complex 3, **E** complex 4, **F** complex 5. Comparisons were made using ANOVA *p ≤ 0.05,**p ≤ 0.01,***p ≤ 0.001,****p ≤ 0.0001

The same mitochondrial function experiments were performed on isolated gastrocnemius mitochondria from all cohorts. O_2_ consumption experiments showed a significant decrease in States 3 and 4u O_2_ consumption in untreated BTHS mice compared to WT controls. BTHS Ex + AAV9 mitochondria showed a trend towards increased O2 consumption in all states and though not significantly improved as compared to BTHS, they were no longer significantly different from WT (Fig. 7A).

We then performed assays to measure the enzyme activity levels of each of the five electron transport chain (ETC) complexes in mitochondria isolated from gastrocnemius muscles of mice from all cohorts. A significant decrease in enzyme activity levels was found in CI, CII, and CIV activities in mitochondria isolated from untreated BTHS gastrocnemius muscles compared to healthy WT controls (Fig. 7B–F). BTHS AAV9 mice showed significant increases in CIII, CIV, and CV activity levels compared to BTHS, and BTHS Ex + AAV9 mice showed significant increases in CI, CIII, CIV, and CV activity levels compared to untreated BTHS controls.

**Fig. 8:**
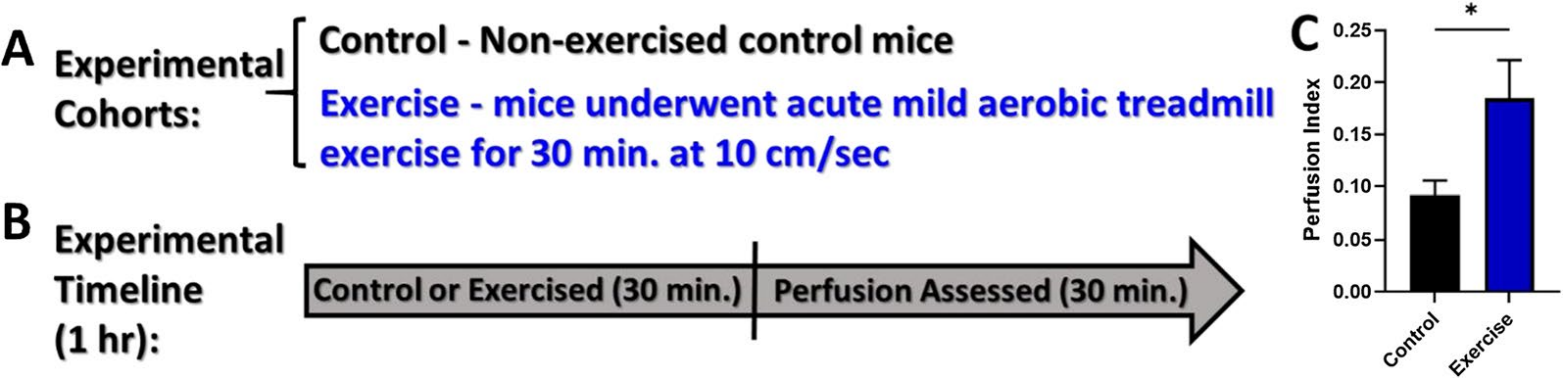
Study to assess perfusion during the 30 min following low intensity aerobic exercise. **A** experimental cohorts, **B** experimental timeline, **C** perfusion indices in WT mice (mean ± std. err. of indices acquired at 0, 5, 10, 15, 20, 25, and 30 min post-intervention) that underwent either no exercise or acute low intensity aerobic exercise. (n = 6 per cohort) *p ≤ 0.05

We hypothesized that one possible explanation for the increased transduction following one round of low-intensity acute exercise may be the altered tissue perfusion. To assess this, mice underwent acute, low intensity, aerobic treadmill exercise (30 min at 10 cm/sec) and were then anesthetized and assessed for the following: temperature, heart rate, oxygen saturation, and perfusion index for an additional 30 min (Fig. 8) using a PhysioSuite multi-physiological monitor. Of all the assessments evaluated, perfusion index (the ratio of the pulsatile blood flow to the non-pulsatile static blood flow in a peripheral tissue—mouse hindlimb paw) was the only parameter that displayed a significant increase in those mice that had undergone acute low-intensity exercise compared to control mice.

## Discussion

Our studies demonstrated that one episode of acute, low-intensity, aerobic exercise immediately prior to systemic AAV9 administration significantly improves AAV uptake by the heart and gastrocnemius and additionally alters uptake in several other tissues. This was demonstrated for the delivery of a marker gene to WT mice and of the transgene *TAFAZZIN* to a mouse model of the cardioskeletal myopathy BTHS. BTHS is an X-linked mitochondrial disorder caused by pathogenic variants in the *TAFAZZIN* gene. BTHS causes a severe cardiomyopathy, muscle weakness, and increased fatigability. No effective, disease-modifying FDA approved treatments for BTHS currently exist, and clinical BTHS standard-of-care includes only palliative administration of medications such as diuretics and ACE inhibitors [22].

The impact of exercise as a factor in AAV distribution has never been directly described. Evidence from a previously published study showed that intramuscular injections of AAV2-*IGF1* into a transgenic mouse model of Amyloid Lateral Sclerosis at 90 days of age that had access to running wheels beginning at 40 days of age, resulted in significant improvements in survival. These results support the combined benefits of AAV gene therapy + exercise for disease treatment in general. However, this study did not consider the direct benefits of exercise on AAV transduction and did not quantify exercise [27]. A muscular dystrophy study tested the hypothesis that voluntary wheel running would complement microdystrophin gene therapy and improve muscle function in a mouse model of DMD [28]. The study clearly demonstrates a benefit to the incorporation of exercise in mice following a gene therapy treatment, which could be a result of the direct benefits of exercise in treated muscles and/or the increased transcription that is activated by continuous exercise. This study did not assess the influence of exercise immediately prior to IV AAV administration. We have observed reduced treadmill fatigability following treatment by AAV-mediated gene therapy, but these tests were performed to evaluate the positive impact of the therapy many weeks post-administration—too late post-administration to be expected to have had a direct influence on AAV transduction [6].

Other acute exercise-focused studies report significant increases in transcription of metabolic and insulin-stimulation pathways and alterations in factors that are important for AAV endocytosis and nuclear trafficking processes (e.g., phosphoinositide 3-kinase/PI3K, HDAC4, and sialic acid modulations) but these did not test AAV delivery [15, 17–19, 29–34]. It is well known that alterations in cell signaling events, insulin metabolism, and cellular receptor translocations occur in response to exercise [35–37]. Future studies will be designed to explore the relative influences of these effects of exercise on AAV efficacy as compared to altered tissue perfusion.

As we work towards the development a clinically relevant gene therapy approach to treat BTHS, we are particularly focused on in improving uptake and therapeutic efficacy in heart and skeletal muscle. The majority, if not all, patients with BTHS are capable of light to moderate exercise [38] and could benefit from an exercise-enhanced gene therapy approach. Specifically, our group previously examined the effect of aerobic and resistance exercise training in adolescents and young adults with BTHS [39, 40]. Both studies found that exercise is safe and tolerated in these individuals [39]. We have also studied the effect of acute exercise on metabolic parameters which showed heightened skeletal muscle metabolism with acute aerobic exercise [41]. In the present study, we found that one episode of low-intensity aerobic exercise led to enhanced *TAFAZZIN* expression, increasingly normalized expression profiles of mitochondrial gene RNA transcripts, and improved mitochondrial function in heart and gastrocnemius samples.

While overall, the trends in tissue expression were similar between the two studies presented here, we observed some slight differences between the spleen and liver expression results from the two different mouse models with C57BL/6 J mice used in the marker gene experiments and a *tafazzin* knockdown mouse model in the BTHS experiments. Of note, all mice in the BTHS experiments were on a doxycycline diet (BTHS affected and healthy control siblings). Although it was not a focus of our investigations, doxycycline is known to bind to serum proteins and can localize in the liver and spleen which may have an impact on vector uptake in these tissues [42]. In addition to the presence or absence of doxycycline and a disease background, another potential contributor to differences observed at the level of transcription would be the use of CMV promoter in the dsRed marker gene experiment and a more tissue restrictive, desmin promoter in the BTHS study.

Our assessments of ETC activities in mitochondria isolated from heart and gastrocnemius showed that in some cases (CI, CIV, and CV for heart and CV for gastrocnemius) the treatment groups had enzyme activity levels that were significantly higher than that found in healthy WT controls. This is likely due to tafazzin’s primary function as a nuclear-encoded transferase that is trafficked to the inner mitochondrial membrane where it remodels monolysocardiolipin (MLCL) to mature cardiolipin (CL). CL is a critical phospholipid involved in maintenance of inner mitochondrial membrane fluidity, osmotic stability, and efficient respiratory chain function and is thought to be particularly important for stabilization of ETC supercomplexes [43–46]. In our previous work we found that IV delivery of our gene therapy vector yielded tafazzin levels that were significantly higher than WT when delivered to adult mice [6]. The effect of tafazzin overexpression on healthy mitochondria has not yet been assessed but it is possible that even within a healthy background, there is capacity to improve ETC enzyme efficiencies.

Our approach is not limited to BTHS as many other monogenic diseases for whom AAV-mediated gene replacement therapies are being developed affects the heart and skeletal muscle. In most of these conditions, exercise is feasible and even recommended as a component of a supportive multidisciplinary care regimen. For example, 70% of boys with Duchenne muscular dystrophy are able to ambulate up to age 10 [47]. Participation in regular, submaximal aerobic activities such as swimming, cycling, muscle strengthening and other lower intensity recreation-based activities are recommended in most individuals with Duchenne [48, 49]. In other neuromuscular and mitochondria associated diseases with a less progressive physical activity decline, regular exercise is not only feasible and safe but improves exercise tolerance, muscle strength, mitochondrial function, and quality of life [39, 50–54]. Thus, many potential human gene therapy recipients are capable of performing some amount of exercise to improve the efficacy of their treatment.

We selected a low intensity aerobic exercise protocol based upon our previous experience with mouse treadmill running and publications from other investigators’ treadmill exercise studies in mice [6, 55, 56]. Our 10 cm/sec speed is within the range considered low (≤ 18 cm/sec) as compared to speeds of ~ 22–25 cm/sec (moderate) or ≥ 40 cm/sec (high) [55, 56]. As BTHS patients and mice are known to demonstrate increased fatigability, we selected a low intensity to ensure the intervention remained within the ability of our BTHS mice to perform. More intense exercise strategies may influence AAV uptake in tissues and this could be tested in future mechanistic studies. However, intense exercise strategies are far less likely to be feasible in patient populations receiving gene therapies.

Acute exercise also alters circulating metabolic signaling cascades, increases gene expression of several mitochondrial and insulin-stimulated pathways, and upregulates cell surface markers [15–19, 57]. In addition to the altered perfusion we observed in this study, these other mechanisms of exercise may also influence AAV uptake and efficacy in a positive way that could potentially be mimicked through a drug strategy. This would provide an enhanced efficacy enhancement option for patients such as infants and those with mobility challenges.

To confirm the generalizability of this discovery, future studies will further analyze the mechanisms of action that underlie this effect, determine the relative merits of different exercise regimens (e.g. mode, intensity, frequency, duration) for specific AAV serotypes or target tissues, and maximize its clinical impact in a variety of therapeutic settings. In addition, large animal studies could confirm the likelihood of success in human populations. In sum, our data suggest that AAV-mediated systemic gene therapies can be optimized without increasing viral doses simply through implementing low-intensity aerobic exercise as a pre-treatment resulting in substantial clinical benefit for monogenic conditions involving the heart and skeletal muscle.

## Methods

### AAV vector design and administration

The MitoTimer plasmid pMitoTimer was a gift from Zhen Yan and used for marker transgene experiments based upon dsRed expression levels (Addgene plasmid # 52659; http://n2t.net/addgene:52659;RRID:Addgene_52659)[58]. It was cloned into a previously described CMV-containing double stranded (ds) AAV plasmid sequence kindly provided by Dr. Xiao Xiao [2]. The full-length human codon optimized *TAFAZZIN* sequence was synthesized by ThermoFisher Gene Art and cloned into a version of Dr. Xiao’s dsAAV containing a previously described desmin (Des) promoter [6, 23]. Each dsAAV plasmid was packaged into recombinant AAV9 capsids that were generated and titered at the University of Florida Vector Core facility in parallel [59]. dsAAV9-CMV-*MitoTimer* or dsAAV9-Des-co*TAFAZZIN* were each intravenously administered to mice at a dose of 5 × 10^12^ vg/kg. Injections were administered through the jugular vein at 6 weeks of age. At 4 weeks post-administration mice were euthanized and necropsies performed to collect fresh tissue for mitochondrial assays. All tissues were also stored at − 80 °C until needed for other assays.

### Mice

The University of Minnesota or the University of Florida IACUC approved all animal studies. Wild type (WT) C57BL/6 J male and female mice were used for the dsAAV9-CMV-MitoTimer study. Wild type (WT) C57BL/6 J female mice were mated to transgenic males (ROSA26 H1/^TetO−shRNA:taz^) CB57BL/129S6 (previously characterized model) for 5 days. Females were then separated from males and placed on a doxycycline (dox) diet containing 200 mg of dox/kg chow (TD98186—Envigo). Transgenic male and female pups were identified by PCR genotyping of tail genomic DNA and maintained on the dox diet throughout their lives. Non-transgenic male and female WT littermates were also maintained on the dox diet and used as controls in BTHS gene therapy experiments.

### Treadmill exercise

Immediately prior to systemic AAV injections, mice were placed on a horizontal ActiTrack v2.7 system treadmill set at 10 cm/sec and exercised for 30 min. Immediately following the 30 min, mice were anesthetized to prepare them for immediate AAV injections as previously described [23, 24].

### Vector genome assessments

A standard curve was generated by spiking in known quantities of either *MitoTimer* plasmid into a background of negative control genomic DNA from WT mice and performing quantitative PCR using *dsRed* primer–probe sets. Genomic DNA was extracted from whole blood and frozen tissues (n = 3–4 per tissue, per treatment cohort) using the Quick-DNA microprep kit (Zymo) and evaluated using the same primer–probe (50 ng of DNA per reaction). Vector genomes were calculated as previously described [3, 60].

### RT-PCR gene expression analyses

Total RNA was isolated from frozen tissues using the RNA Extraction kit (Zymo). cDNA was synthesized using the High Capacity RNA-to-cDNA kit (Applied Biosystems) and quantitative real-time (RT)-PCR was performed using TaqMan Master Mix (Thermo Scientific) and transgene specific primers (Thermo Scientific) on a StepOnePlus Real-Time PCR System (Applied Biosystems). Relative expression levels were calculated using the ΔΔCt method to determine fold increase compared to control levels.

### Profiler arrays

Arrays containing 84 mitochondrial genes, 5 housekeeping genes, and experimental control wells (RT^2^ Profiler™ PCR Array Mouse Mitochondria—PAMM-087Z—Qiagen) were used to compare cohorts in the BTHS study. RNA from either heart or gastrocnemius samples was reverse transcribed into cDNA using the RT^2^ First Strand Kit (Qiagen) and assessed using on a StepOnePlus Real-Time PCR System (Applied Biosystems) using RT^2^ SYBR Green qPCR MasterMix (Qiagen). Shiny GO 0.77 was used for gene ontology analyses of biological processes [61].

### Oxytherm O_2_ consumption measurements

Mitochondrial isolations were performed on fresh tissue as previously described [62]. Oxygen consumption measurements were acquired by diluting samples in 0.5 mL of respiration buffer (Mir05—0.5 mM EGTA, 3 mM MgCl_2_, 60 mM lactobionic acid, 20 mM taurine, 10 mM KH_2_PO_4_, 20 mM HEPES, 110 mM sucrose, 1 g/l BSA) in the chamber of an Oxytherm electrode unit (Hansatech, Norfolk, UK) at 37 °C with constant stirring speed (60 rpm). The probe electrodes were calibrated with sodium dithionite (0% oxygen) and respiration buffer (100% oxygen) as recommended by the manufacturer. The measurements were acquired following additions of: 100 µL of isolated mitochondria, 10 µL substrate (0.25 M Glutamate/0.125 M Malate), 7.5 µL 10 mM Adenosine 5’-diphosphate sodium salt (ADP) (State 3), 0.5 µL of oligomycin (5 mg/mL) (State 4_o_), and 1 µL of 100 µM Carbonyl cyanide 4-(trifluoromethoxy) phenylhydrazone (FCCP) (State 4_u_).

### OxPhos. Electron transport chain complex activity assays (CI, CII, CIII, CIV, CV)

Briefly, CI activity was determined by oxidation of NADH to NAD at 340 nm using a SpectraMax i3x. 10 µL of isolated mitochondrial homogenate was diluted into 90 µL of 50 mM phosphate buffer (pH 7.5), 100 µM NADH, 60 µM ubiquinone, 3 mg/mL fatty acid-free bovine serum albumin (BSA), 300 µM potassium cyanide (KCN), and 10 µM rotenone (in parallel). CI activity was described as % inhibition following addition of rotenone. CII activity was determined by reduction of DCPIP (2,6-diclorophenolindophenol sodium salt hydrate) at 600 nm. 10 µL of isolated mitochondrial homogenate was diluted into 90 µL of 25 mM phosphate buffer (pH 7.5), 1 mg/mL fatty acid-free BSA, 300 µM KCN, 20 mM succinate, 50 µM decylubiquinone, 80 µM DCPIP, and 10 mM malonate (in parallel). CII activity was described as % inhibition following addition of malonate. CIII activity was measured as the conversion of oxidized cytochrome c to a reduced form at 550 nm. 10 µL of mitochondrial homogenate was diluted into 90 µL of 25 mM phosphate buffer (pH 7.5), 75 µM oxidized cytochrome c, 500 µM of KCN, 100 µM EDTA (pH 7.5), 0.025% (vol/vol) Tween-20, 100 µM of decylubiquinol and 10 µg/mL antimycin A (in parallel). CIII activity was described as % inhibition following the addition of antimycin A. CIV activity was determined by oxidation of reduced Cytochrome C. 5 µL of isolated mitochondria was diluted into 95 µL of 25 mM phosphate buffer (pH 7.0), 50 µM reduced cytochrome c and 300 µM KCN (in parallel). CIV activity was described as % inhibition following the addition of KCN. CV activity was determined using the ATPlite Luminescence Assay System (PerkinElmer) as directed by the manufacturer. Activity data for all experimental cohorts are displayed relative to WT activities set to 100%.

### Mouse physiosuite vitals assessments

Perfusion index, heart rate, temperature, and O_2_ saturation were acquired using the PhysioSuite instrument for mice (Kent Scientific). Immediately following completion of the 30 min low intensity aerobic exercise, mice were anesthetized. Vitals were measured on the anesthetized animals for 30 min. A soft-touch paw sensor was attached to the left hind-limb paw to measure perfusion index (the ratio of the pulsatile blood flow to the non-pulsatile static blood flow in a peripheral tissue), heart rate, and O_2_ saturation. A rectal thermometer measured temperature.

### Statistical analyses

Data were analyzed using GraphPad Prism Software. Values are reported as mean ± standard error. Significant differences were determined by t-tests or one way ANOVA (* p ≤ 0.05, ** p ≤ 0.01, *** p ≤ 0.001, **** p ≤ 0.0001 were considered statistically significant).

## Data Availability

Data and materials will be made available upon request.
